# Immunologic, Anti-Inflammatory, and Anti-Muscle Damage Profile of Supplemented Vitamin D_3_ in Healthy Adults on Strenuous Endurance Exercise

**DOI:** 10.3390/biology12050657

**Published:** 2023-04-26

**Authors:** Ming-Che Liu, Pei-Wei Weng, Sheng-Chang Chen, Ting-Hao Liu, Hsiang-Wei Huang, Chang-Ti Huang, Cheng-Tse Yang, Viraj Krishna Mishra, Ming-Ta Yang

**Affiliations:** 1 School of Dental Technology, Taipei Medical University, Taipei 110301, Taiwan; 2 Clinical Research Center, Taipei Medical University Hospital, Taipei 110301, Taiwan; 3 Department of Urology, Taipei Medical University Hospital, Taipei 110301, Taiwan; 4 Graduate Institute of Clinical Medicine, Taipei Medical University, Taipei 110301, Taiwan; 5 Research Center of Urology and Kidney, Taipei Medical University, Taipei 110301, Taiwan; 6 School of Medicine, Taipei Medical University, Taipei 110301, Taiwan; 7 Department of Orthopaedics, Shuang Ho Hospital, Taipei Medical University, New Taipei City 235041, Taiwan; 8 International Ph.D. Program in Biomedical Engineering, Taipei Medical University, Taipei 110301, Taiwan; 9 Center for General Education, Taipei Medical University, Taipei 110301, Taiwan; 10 Department of Medical Education, E-DA Hospital, Kaohsiung 824005, Taiwan; 11 Graduate Institute of Medical Sciences, Taipei Medical University, Taipei 110301, Taiwan; 12 BioSource Tech, Ambala 133101, Haryana, India

**Keywords:** strenuous endurance exercise, CD4^+^/CD8^+^ ratio, inflammation, creatine kinase, heart rate, Interleukin-6, lactate dehydrogenase

## Abstract

**Simple Summary:**

Vitamin D_3_ may regulate inflammation, immunological responses, and muscle damage; however, its impact on these physiological activities remains obscure. This study reported the effects of vitamin D_3_ supplementation on immune response, inflammatory profile, muscle damage, and aerobic capacity. The participants received either 5000 IU of vitamin D_3_ or placebo for 4 weeks. The time to exhaustion, maximal heart rate, and average heart rate were also recorded during the exercise test. Venous blood samples were drawn before, immediately after, and 2, 4, and 24 h after exercise to assess total and differential blood leukocyte counts, levels of cytokines, and muscle damage biomarkers. We found that daily 5000 IU vitamin D_3_ supplementation for 4 weeks may effectively increase blood 25(OH)D levels, immune response, and aerobic capacity while inhibiting inflammatory cytokines and muscle damage in people performing strenuous endurance exercise. Vitamin D_3_ seems to be a useful nutritional strategy to attenuate immune suppression, inflammatory responses, and muscle damage in people performing an intensive exercise.

**Abstract:**

Reportedly, strenuous endurance exercise can depress the immune system and induce inflammation and muscle damage. Therefore, this double-blinded, matched-pair study aimed to investigate the impact of vitamin D_3_ supplementation on immune response (leukocyte, neutrophil, lymphocyte, CD4^+^, CD8^+^, CD19^+^, and CD56^+^ counts), inflammatory profile (TNF-α and IL-6), muscle damage (CK and LDH levels), as well as aerobic capacity after strenuous endurance exercise in 18 healthy men taking 5000 IU of vitamin D_3_ (*n* = 9) or placebo (*n* = 9) daily for 4 weeks. Total and differential blood leukocyte counts, levels of cytokines, and muscle damage biomarkers were determined before, immediately after, and 2, 4, and 24 h after exercise. The IL-6, CK, and LDH levels were significantly lower in vitamin D_3_ group at 2, 4, and 24 h post exercise (*p* < 0.05). Maximal and average heart rates during exercise were also significantly lower (*p* < 0.05). In the vitamin D_3_ group, the CD4^+^/CD8^+^ ratio after 4 weeks of supplementation was only significantly lower at post-0 than at baseline and significantly higher at post-2 than at baseline and post-0 (all *p* < 0.05). Taken together, 5000 IU of daily vitamin D_3_ supplementation for 4 weeks exhibited positive effects in terms of increased blood 25(OH)D levels, CD4^+^/CD8^+^ ratio (immune response), and aerobic capacity while inhibiting inflammatory cytokines and CK and LDH (muscle damage) in people performing strenuous endurance exercise.

## 1. Introduction

An increasing body of evidence implies that despite various beneficial attributes, certain types of exercise may impose significant physiological stresses. In particular, repeated bouts of strenuous endurance exercise have been associated with suppressed immune function, inflammation, and muscle damage [1,2]. The immune system prevents infection and is involved in the maintenance of tissue repair, metabolism, sleep, fatigue, and mental health systems [3]. Notably, studies have demonstrated muscle damage events initiated even within 24 h through inflammatory events. Specifically, in the early hours of the recovery period, neutrophils dominate the inflammatory cell profile, acting to clear cellular debris and propagating cytokine-secreted inflammatory response [2]. Further, mast cells also infiltrate muscle tissue, releasing histamine and chemo-attractants. Within 4 to 24 h after muscle damage, proinflammatory macrophages infiltrate the muscle and release proinflammatory cytokines. These macrophages also remove damaged tissue through phagocytosis and stimulate the proliferation of myoblasts. After 24 h, proinflammatory macrophages are replaced by anti-inflammatory macrophages and CD8^+^ and T-regulatory lymphocytes. These cells secrete anti-inflammatory cytokines, recruit macrophages, and stimulate myoblast proliferation and expansion of the satellite cell pool.

Studies have investigated the incidence of upper respiratory tract infection (URTI) and cellular immunity after high-intensity exercise [4,5]. Lymphocytes are cellular immunity components that are divided into helper T (CD4^+^), cytotoxic T (CD8^+^), B (CD19^+^), and natural killer (NK; CD56^+^) cells. Notably, strenuous exercise could reduce the CD4^+^/CD8^+^ ratio and therefore may represent a relevant marker of immunological change [6,7,8,9]. A decrease in CD4^+^, CD8^+^, CD19^+^, and CD56^+^ lymphocytes during the recovery period (1–2 h) post high-intensity exercise indicates the immunosuppressive effect, which is also known as an open-window impact [3,10,11]. Inflammation is a primary immune response involved in the prevention of infection, damage, and noxious conditions for the maintenance of tissue repair and homeostasis [12,13]. Reportedly, inflammation-related markers such as tumor necrosis factor-α (TNF-α) and interleukin (IL)-6 have been found to have considerably increased post strenuous exercise [14,15,16]. Remarkably, strenuous exercise generates free radicals, reactive oxygen (ROS), and nitrogen species in tissues and cells [17]. Further, high-intensity exercise could destroy tissues, damage cells, and inhibit muscle growth along with an increase in creatine kinase (CK) and lactate dehydrogenase (LDH) levels [18].

Vitamin D, an essential fat-soluble vitamin, occurs in two main forms: ergocalciferol (D_2_), obtained from vegetables or supplements, and cholecalciferol (D_3_) [19,20]. Vitamin D_3_ synthesis is triggered in the skin after ultraviolet B exposure. Additionally, it could be acquired from oily fish, fortified food, and oral supplements, and according to an International Olympic Committee statement in 2018, vitamin D_3_ supplements among athletes could promote immune health and assist with training, recovery, muscle soreness, and injury management [21]. However, vitamin D deficiency is common among the general public as well as in athletes worldwide [22,23,24,25,26]. Vitamin D deficiency leads to mitochondrial dysfunction, decreased adenosine triphosphate (ATP) production, increased ROS production, oxidative damage, muscle atrophy, and impaired muscle function [3,4]. Remarkably, activated (hydroxylated) vitamin D [1,25(OH)D] stimulates an abundance of vitamin D receptors (VDR) in satellite cells and central myonuclei during muscle regeneration. Moreover, vitamin D inhibits B-cell proliferation, differentiation, and immunoglobulin secretion [27]. In addition, T-cell proliferation is also inhibited, indicating a resultant shift from a Th1 to a Th2 phenotype [27,28]. Notably, the inhibition of Th17 cell development and facilitation of T regulatory cells increases the production of anti-inflammatory cytokines such as IL-10 while decreasing the production of inflammatory cytokines (IL-17 and IL-21) [29,30,31,32]. Vitamin D also inhibits the monocyte production of inflammatory cytokines such as TNF-α, IL-1, IL-6, IL-8, and IL-12 [33].

In one study, a 14-week vitamin D_3_ supplementation (at 5000 IU/day) in athletes led to an increase in blood 25(OH)D levels and ameliorated immune function [34]. Similarly, vitamin-D-deficient taekwondo athletes on 4-week vitamin D_3_ supplementation (at 5000 IU/day) demonstrated improved immune health through significantly elevated 25(OH)D levels and a decrease in URTI risk [35]. Notably, several studies have revealed mixed results on the effects of vitamin D supplementation on inflammatory cytokines and muscle damage [36,37,38,39]. Specifically, some studies indicated a reduction in the inflammatory response and muscle damage [36,38], whereas others reported the absence of significant differences [37,39]. Thus, the above literature suggests that vitamin D_3_ may regulate inflammation, immunological responses, and muscle damage. However, the conclusion on the impacts of vitamin D on the maintenance and restoration of muscle damage/strength and inflammation remains obscure. Therefore, we hypothesize that the outcomes of our study will help in reaching a clear consensus on the impact of vitamin D_3_ supplementation on immune response, inflammatory cytokine production, and muscle damage after strenuous endurance exercise. We determined the levels of CD4^+^/CD8^+^, CD19^+^, CD56^+^, leukocytes, neutrophils, and lymphocytes for assessing immune response. The inflammatory response was estimated through TNF-α and IL-6, while muscle damage creatine kinase (CK) and lactate dehydrogenase (LDH) represented muscle damage. Additionally, we measured blood 25(OH)D, maximal heart rate (HR_max_), average heart rate (AHR), and maximal oxygen consumption (VO_2max_) to assess the extent of vitamin D_3_’s impact on aerobic capacity. We anticipate that the finding of this study will contribute to determining the role of vitamin D_3_ supplementation to improve the quality of life and performance during strenuous endurance exercise.

## 2. Materials and Methods

### 2.1. Participants

Using G*Power software (v.3.1.9.2) and a 2 × 2 mixed-design analysis of variance (ANOVA) with an effect size of 0.4, an alpha error of 0.05, and a power of 0.80, the total number of subjects was calculated to be 16; however, 18 subjects were finally recruited above the cut-off value. Individuals with diabetes or cardiovascular, liver, renal, or autoimmune diseases; blood 25(OH)D levels > 30 ng/mL; or maximal oxygen consumption VO_2max_ < 40 mL/kg/min were excluded. None of the included participants regularly consumed supplements during the experimental period. Participants were requested to maintain a regular lifestyle and avoid the consumption of alcohol and other nutritional supplements. All participants were asked to complete the study between March and May 2021 to minimize variability due to UV exposure. The participant characteristics are listed in Table 1.

### 2.2. Experimental Design and Procedure

**Figure 1:**
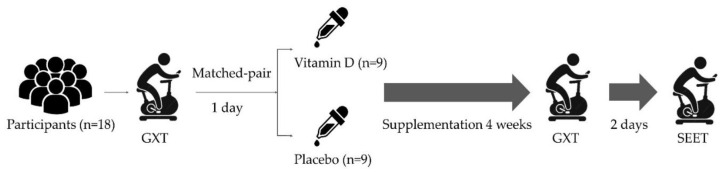
Experimental scheme. GXT, graded exercise test; SEET, strenuous endurance exercise test.

Figure 1 illustrates the schematic representation of the present study. All participants performed a graded exercise test (GXT) until exhaustion on a cycle ergometer to determine VO_2max_ 1 day before supplementation. A double-blinded, matched-pair design was adopted to divide all 18 participants into the vitamin D_3_ (*n* = 9) and placebo (*n* = 9) groups based on their VO_2max_ level. The VO_2max_ was measured post 4 weeks of supplementation. A strenuous endurance exercise test (SEET) was conducted 2 days after the GXT, and blood samples were collected before (baseline), immediately after (post-0), and 2 h (post-2), 4 h (post-4), and 24 h after (post-24) exercise. Immune response biomarkers (i.e., leukocyte, neutrophil, and lymphocytes (CD4^+^, CD8^+^, CD19^+^, and CD56^+^) counts and inflammatory cytokines (TNF-α and IL-6 levels)) were analyzed at pre, post-0, post-2, post-4, and post-24, while muscle damage biomarkers (i.e., CK and LDH levels) were analyzed at pre, post-0, post-4, and post-24. The time to exhaustion, maximal heart rate (HR_max_), and average heart rate (AHR) were also recorded during the exercise test.

### 2.3. Vitamin D_3_ Supplementation

The supplementation protocol and dosage were applied according to the method explained in another study [40]. During the experiment period, individuals in the supplementation group were asked to consume 5000 IU of vitamin D_3_ in the oil form (Liquid Shield Vitamin D_3_+E; Panion & BF Biotech, Taipei, Taiwan) after lunch for 4 weeks. The placebo group received medium-chain triglycerides (Panion & BF Biotech). The supplements provided to both groups had identical taste and color.

### 2.4. GXT Protocol

The current GXT protocol, based on another study [41], was used to determine the intensity of the exercise test (65% VO_2max_) on a cycling ergometer (Monark LC6; Monark Exercise AB, Sweden). After a warm-up for 5 min at 50 W (velocity = 70 ± 5 rpm), the workload was increased by 25 W every 2 min. Strong verbal encouragement was provided to the participants to maintain 70 rpm speed. The test was terminated when the participants were not able to maintain at least 60 rpm. Exchange volume, VO_2_, and VCO_2_ were measured using gas analysis (MetaMax 3B; Cortex, Leipzig, Germany). In addition, heart rate (S610; Polar, Kempele, Finland) was also measured simultaneously. Each participant’s rating of perceived exertion (RPE) was also recorded during every stage on a standard Borg scale [42]. Individual VO_2max_ was achieved when at least two of the following criteria were met: (1) heart rate ≤ 15 beats/min of individual predicted HR_max_, (2) RPE > 18, and (3) respiratory exchange ratio > 1.1 [43].

### 2.5. Strenuous Endurance Exercise Test (SEET)

SEET was conducted 2 days after the participants completed the GXT. The participants rested for 30 min before the test and wore a Polar 610 to monitor their heart rates. Participants exercised at 65% VO_2max_ for 2 h after a 5 min warm-up to a heart rate of 150 beats/min. Based on the participants’ heart rate and VO_2max_, the workload of the cycle ergometer was adjusted to maintain the demand intensity of effort. After a 2 h cycling exercise at 70 rpm, we verbally encouraged the participants to increase their exercise speed to 100 rpm until exhaustion at the present workload (defined by the failure to maintain a speed of ≥90 rpm). Their oxygen consumption, AHR, and HR_max_ were recorded. During the test, the participants were provided with ≤300 mL of water every 15 min if needed. The criteria of volitional exhaustion were identical to the evaluation criteria for VO_2max_.

### 2.6. Biochemical Variables

Blood samples (1 mL) were collected through the antecubital vein before and after vitamin D_3_ supplementation and centrifuged at 3000 rpm for 10 min at 4 °C (Centrifuge 5702 R; Eppendorf, Hamburg, Germany). The method of 25(OH)D analysis was taken from another study [44]. Furthermore, blood specimens (10 mL) were drawn at baseline, post-0, post-2, post-4, and post-24 through the antecubital vein in two dry vacutainer tubes (one with ethylenediaminetetraacetic acid and the other with clot activator) to analyze leukocyte, neutrophil, lymphocyte, CD4^+^, CD8^+^, CD19^+^, and CD56^+^ counts along with TNF-α, IL-6, CK, and LDH levels. Subsequently, a small whole-blood sample (50 μL) was used to examine leukocyte, neutrophil, and lymphocyte counts by hematology analyzer (Sysmex XP300, Chuo-ku, Kobe, Japan).

CD4^+^, CD8^+^, CD19^+^, and CD56^+^ counts were measured using PE-Cy™7 Mouse Anti-Human CD4 (Cat. No. 557852), PerCP-Cy™5.5 Mouse Anti-Human CD8 (Cat No. 565310), PE Mouse Anti-Human CD19^+^ (Cat. No. 555413), and BB515 Mouse Anti-Human CD56^+^ (Cat. No. 564488), respectively (all from BD Biosciences, San Jose, CA, USA), according to the manufacturer’s instructions. First, 200 μL whole blood was transferred to a conical polypropylene test tube, and then, 2000 μL of lysing buffer was added and incubated at room temperature for 15 min. The solution was centrifuged at 1200 rpm for 5 min, and the supernatant was discarded. Next, 2000 μL of stain buffer was added, followed by centrifugation at 1200 rpm and 4 °C for 5 min; the supernatant was discarded again. Thereafter, 100 μL of stain buffer, 1 μL of CD4^+^ antibody, 1 μL of CD8^+^ antibody, 4 μL of CD19^+^ antibody, and 2 μL of CD56^+^ antibody was added, and the solution was incubated on ice in the dark for 45 min. Next, 900 μL of stain buffer was added, followed by centrifugation at 1200 rpm and 4 °C for 5 min; the supernatant was discarded. Finally, 300 μL of stain buffer was added, and the sample was analyzed on a BD FACS Calibur flow cytometer (Becton Dickson, San Jose, CA, USA). For the gating strategies, initially, the forward and side scatter characteristics were used to gate lymphocytes. After the acquisition of 20,000 lymphocyte events, CD4^+^, CD8^+^, CD19^+^, and CD56^+^ cells were then gated by the surface expression of respective antibodies.

Other blood samples were centrifuged at 3000 rpm and 4 °C for 10 min. Serum CK and LDH levels were analyzed on Cobas c702 (Roche Diagnostics, Basel, Switzerland). The plasma was collected in microcentrifuge tubes and stored at −80 °C before analysis for TNF-α and IL-6 levels by using commercial enzyme-linked immunosorbent assay kits from R&D Systems (Minneapolis, MN, USA), according to the manufacturer’s instructions, with inter-assay coefficients of variance of <6.7% and <10.8%, respectively.

### 2.7. Statistical Analysis

Data are expressed as means ± standard deviations. An independent-sample *t*-test was used to compare the participant characteristics and aerobic capacity between the groups. A 2 (groups: vitamin D_3_ and placebo) × 2 (time points: before and after supplementation) two-way, mixed-design analysis of variance (ANOVA) was used to compare the levels of 25(OH)D, immune response biomarkers, inflammatory cytokines, and muscle damage biomarkers. Significant main effects of the group were further analyzed using independent *t*-tests. The main effect of the time was also further analyzed using the paired *t*-tests to examine the differences in 25(OH)D levels before and after supplementation. We also used one-way repeated-measures ANOVA and the least significant difference method to examine the differences in immune responses, inflammatory cytokines, and muscle damage among time points. The Hedge’s g values were used to interpret the small (0.20–0.49), moderate (0.50–0.79), or large (≥0.80) effect size [45]. Post hoc independent *t*-tests were performed on the main effects of the group. All statistical analyses were performed using IBM SPSS (version 22.0; IBM, Armonk, NY, USA). The significance level was set to *p* < 0.05.

## 3. Result

### 3.1. Blood 25(OH)D Levels

**Figure 2:**
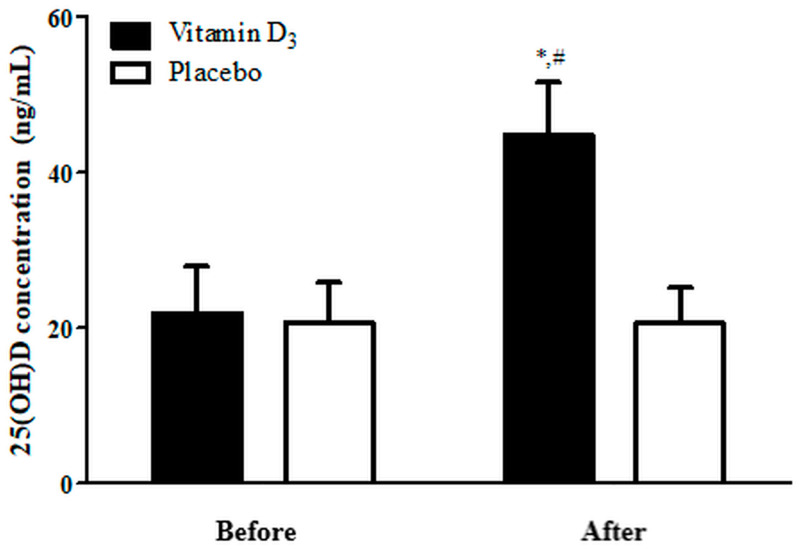
Blood 25(OH)D levels before and after 4-week supplementation. * *p* < 0.05 compared with before supplementation. # *p* < 0.05 compared with placebo.

Figure 2 illustrates blood 25(OH)D levels before and after 4-week vitamin D_3_ and placebo supplementation. A two-way, mixed-design ANOVA with group and time indicated the interaction between two factors was significant (*p* < 0.05). The 25(OH)D levels in the placebo group exhibited no significant differences before and after supplementation. Nevertheless, in the vitamin D_3_ group, 25(OH)D levels after 4-week supplementation were significantly higher than the placebo group and before supplementation (*p* < 0.05; Hedge’s g = 1.00). These findings indicate that our supplementation strategy effectively increased blood 25(OH)D levels.

### 3.2. Effects of Vitamin D_3_ Supplementation on Immune Response

**Figure 3:**
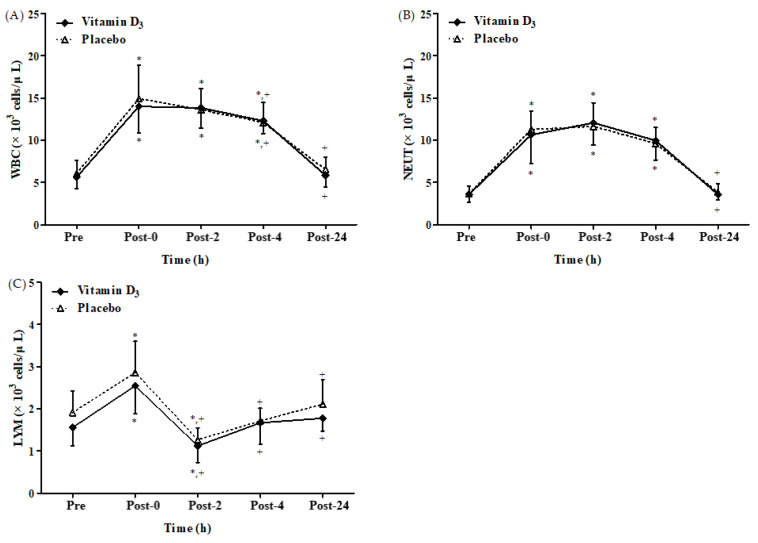
Changes in (**A**) leukocyte, (**B**) neutrophil, and (**C**) lymphocyte counts after strenuous endurance exercise post 4-week vitamin D_3_ supplementation. * *p* < 0.05 compared with baseline. *+ p* < 0.05 compared with post-0. WBC, leukocyte; NEUT, neutrophil; LYM, lymphocyte; Pre, baseline (pre-exercise); Post-0, immediately after exercise; Post-2, 2 h after exercise; Post-4, 4 h after exercise; Post-24, 24 h after exercise.

A significant time main effect was observed on leukocyte, neutrophil, and lymphocyte counts after 4-week vitamin D_3_ or placebo supplementation, respectively (Figure 3A–C). There was no significant difference between groups and no significant interaction between the effects of group and time. Figure 3A,B present leukocyte and neutrophil counts. Figure 3A,B present leukocyte and neutrophil counts after 4-week vitamin D_3_ or placebo supplementation, respectively. Both counts were significantly higher at post-0, post-2, and post-4 than at baseline in both groups (all *p* < 0.05). However, the counts returned to baseline levels in both groups at post-24. Figure 3C illustrates lymphocyte counts after a 4-week vitamin D_3_ or placebo supplementation. They were significantly higher at post-0 than at baseline in both groups (both *p* < 0.05). Moreover, the lymphocyte counts were significantly lower at post-2 than at baseline and post-0 (all *p* < 0.05) in both groups. Taken together, these results indicate that strenuous endurance exercise induces immune suppression and inflammatory response.

**Figure 4:**
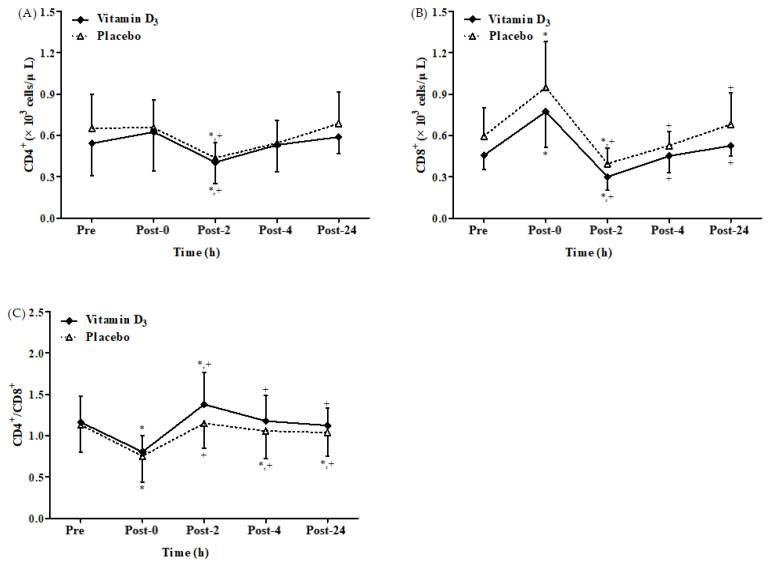
Changes in (**A**) CD4^+^ and (**B**) CD8^+^ count as well as (**C**) CD4^+^/CD8^+^ ratio after strenuous endurance exercise post 4-week vitamin D_3_ supplementation. * *p* < 0.05 compared with baseline. + *p* < 0.05 compared with post-0. Pre, baseline (pre-exercise); Post-0, immediately after exercise; Post-2, 2 h after exercise; Post-4, 4 h after exercise; Post-24, 24 h after exercise.

There was a significant time main effect on CD4^+^ and CD8^+^ counts as well as CD4^+^/CD8^+^ ratio after 4-week vitamin D_3_ or placebo supplementation, respectively (Figure 4A–C). There was no significant difference between groups and no significant interaction between the effects of group and time. Figure 4A,B present CD4^+^ and CD8^+^ count after 4-week vitamin D_3_ or placebo supplementation, respectively. In both groups, the CD4^+^ counts were significantly lower at post-2 than at baseline and post-0 (all *p* < 0.05). However, the differences in CD4^+^ counts at the other time points were insignificant in both groups. The CD8^+^ counts in both the groups were significantly higher at post-0 than at baseline and significantly lower at post-2 than at baseline and post-0 (all *p* < 0.05). Figure 4C presents the CD4^+^/CD8^+^ ratios after 4-week vitamin D_3_ supplementation or placebo. In the placebo group, the CD4^+^/CD8^+^ ratio was significantly lower at post-0, post-4, and post-24 than at baseline (all *p* < 0.05), but the differences in the CD4^+^/CD8^+^ ratio between post-2 and baseline were insignificant. However, in the vitamin D_3_ group, the CD4^+^/CD8^+^ ratio after 4 weeks of supplementation was only significantly lower at post-0 than at baseline and significantly higher at post-2 than at baseline and post-0 (all *p* < 0.05). However, the difference in the CD4^+^/CD8^+^ ratio in the vitamin D_3_ group between post-4 and baseline and between post-24 and baseline was insignificant. These results indicate that 4-week vitamin D_3_ supplementation has positive effects on the immune system when the open window is induced by strenuous endurance exercise.

**Figure 5:**
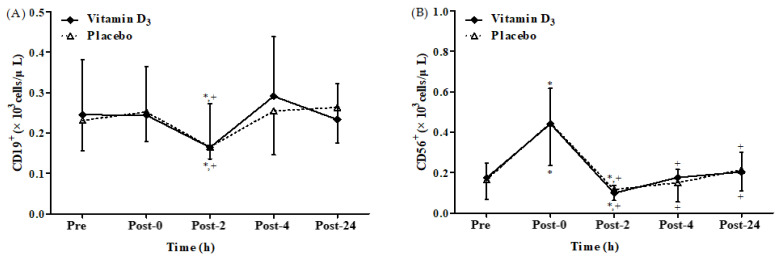
Changes in (**A**) CD19^+^ and (**B**) CD56^+^ counts after strenuous endurance exercise post 4-week vitamin D_3_ supplementation. * *p* < 0.05 compared with baseline. + *p* < 0.05 compared with post-0. Pre, baseline (pre-exercise); Post-0, immediately after exercise; Post-2, 2 h after exercise; Post-4, 4 h after exercise; Post-24, 24 h after exercise.

We found a significant time main effect on CD19^+^ and CD56^+^ counts after 4-week vitamin D_3_ or placebo supplementation, respectively (Figure 5A,B). However, no significant difference between groups and no significant interaction between the effects of group and time were observed. Figure 5A,B present CD19^+^ and CD56^+^ counts after 4-week vitamin D_3_ or placebo supplementation, respectively. In both groups, the CD19^+^ counts were significantly lower at post-2 than at baseline and post-0 (all *p* < 0.05). However, the counts in both groups also exhibited no significant difference between post-4 and baseline or between post-24 and baseline. In both groups, the CD56^+^ counts were significantly higher at post-0 than at baseline (*p* < 0.05) but significantly lower at post-2 than at baseline and post-0 (all *p* < 0.05). These results revealed that vitamin D_3_ supplementation does not affect CD19^+^ and CD56^+^ counts after strenuous endurance exercise.

### 3.3. Effects of Vitamin D_3_ Supplementation on Inflammatory Cytokines

**Figure 6:**
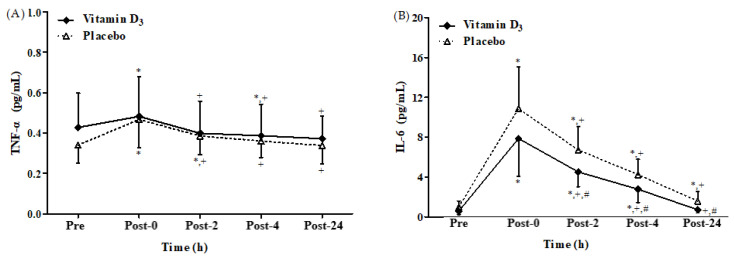
Changes in (**A**) TNF-α and (**B**) IL-6 levels after strenuous endurance exercise post 4-week vitamin D_3_ supplementation. * *p* < 0.05 compared with baseline. + *p* < 0.05 compared with post-0. # *p* < 0.05 compared with placebo. TNF-α, tumor necrosis factor-α; IL-6, inter-leukin-6; Pre, baseline (pre-exercise); Post-0, immediately after exercise; Post-2, 2 h after exercise; Post-4, 4 h after exercise; Post-24, 24 h after exercise.

A significant time main effect was detected, with no significant difference between groups and no significant interaction between the effects of group and time on the TNF-α level (Figure 6A). Figure 6A,B present TNF-α and IL-6 levels after 4-week vitamin D_3_ or placebo supplementation, respectively. In the placebo group, TNF-α levels were significantly higher at post-0 and post-2 than at baseline (both *p* < 0.05). Similarly, significant group and time main effects, with no significant interaction between the effects of group and time on the IL-6 level (Figure 6B), were observed. However, in the vitamin D_3_ group, they were only significantly higher at post-0 than at baseline (*p* < 0.05). In addition, TNF-α levels were significantly lower at post-4 than at baseline in the vitamin D_3_ group (*p* < 0.05). IL-6 levels were significantly higher at post-0, post-2, post-4, and post-24 than at baseline in the placebo group (all *p* < 0.05). However, IL-6 levels were significantly higher only at post-0, post-2, and post-4 than at baseline in the vitamin D_3_ group (all *p* < 0.05). Moreover, IL-6 levels were significantly lower at post-2, post-4, and post-24 in the vitamin D_3_ group than the placebo group (all *p* < 0.05; Hedge’s g = 0.73, 0.66, and 0.77). Therefore, vitamin D_3_ supplementation exhibited a suppressed inflammatory response after strenuous endurance exercise.

### 3.4. Effects of Vitamin D_3_ Supplementation on Muscle Damage

**Figure 7:**
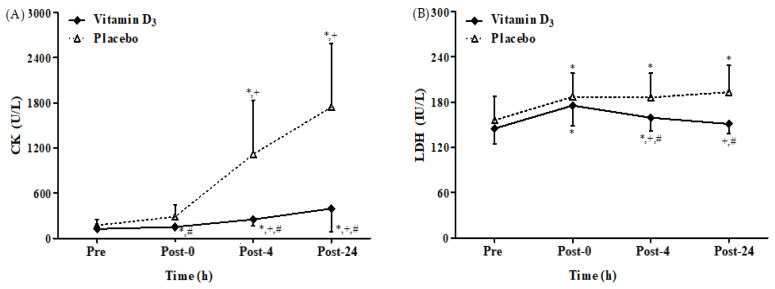
Changes in (**A**) CK and (**B**) LDH levels after strenuous endurance exercise post 4-week vitamin D_3_ supplementation. * *p* < 0.05 compared with baseline. + *p* < 0.05 compared with post-0. # *p* < 0.05 compared with placebo. CK, creatine kinase; LDH, lactate dehydrogenase; Pre, baseline (pre-exercise); Post-0, immediately after exercise; Post-4, 4 h after exercise; Post-24, 24 h after exercise.

We found a significant interaction between the effects of group and time on CK and LDH levels. Figure 7A,B present CK and LDH levels after 4-week vitamin D_3_ or placebo supplementation. The CK levels in both groups were significantly higher at post-4 and post-24 than at baseline and post-0 (all *p* < 0.05). In addition, they were significantly lower at post-0, post-4, and post-24 in the vitamin D_3_ group than the placebo group (all *p* < 0.05; Hedge’s g = 0.75, 0.96, and 1.00). The LDH levels were significantly higher at post-0, post-4, and post-24 than at baseline in the placebo group (all *p* < 0.05). However, in the vitamin D_3_ group, the LDH levels were significantly higher only at post-0 and post-4 than at baseline (*p* < 0.05) but significantly lower at post-4 and post-24 than at post-0 (all *p* < 0.05). Moreover, the LDH levels were significantly lower at post-4 and post-24 in the vitamin D_3_ group than the placebo group (*p* < 0.05; Hedge’s g = 0.67 and 0.95). These results indicate that vitamin D_3_ supplementation can effectively attenuate strenuous endurance exercise-induced muscle damage.

### 3.5. Impact of Vitamin D_3_ Supplementation on Aerobic Capacity

All participants completed strenuous endurance exercises, including cycling at a 65% VO_2max_ intensity for 2 h on a cycle ergometer. HRmax and AHR during exercise were significantly lower in the vitamin D_3_ group than in the placebo group (both *p* < 0.05; Hedge’s g = 0.79 and 0.80; Table 2). However, no significant difference between the group in the time to exhaustion and VO_2max_ (both *p* > 0.05) was observed. These results indicate that 4-week vitamin D_3_ supplementation increases aerobic capacity by reducing heart rate.

## 4. Discussion

In the current study, we investigated the effects of 4-week vitamin D_3_ supplementation on immune response (leukocyte, neutrophil, lymphocyte, CD4^+^, CD8^+^, CD19^+^, and CD56^+^ counts), inflammatory cytokines (TNF-α and IL-6 levels), muscle damage (CK and LDH levels), and cardiovascular capacity (time to exhaustion, HR_max_, AHR, and VO_2max_). To the best of our knowledge, this is the first study to examine the effects of vitamin D_3_ supplementation on immune suppression, inflammatory response, muscle damage, and aerobic capacity after strenuous endurance exercise. Our results indicate that 4-week vitamin D_3_ supplementation resulted in an enhanced aerobic capacity and CD4^+^/CD8^+^ ratio as well as lowered IL-6, CK, and LDH levels through performed strenuous endurance exercises. This result is consistent with those of studies indicating that 4-week vitamin D_3_ supplementation (at 5000 IU/day) can effectively enhance blood 25(OH)D levels—ranging from insufficient (21.89 ng/mL) to sufficient (44.67 ng/mL; Figure 2) [46,47].

In the present study, the lymphocyte counts were higher at post-0, and they decreased at post-2 to levels significantly lower than those at baseline (Figure 3C). Therefore, executing the 2 h strenuous endurance exercise at 65% VO_2max_ can induce the open window, which is consistent with the results of other reports [11,48]. Elevated lymphocyte levels at post-0 were caused by lymphocyte recruitment [49]; the mechanism underlying lymphocyte recruitment includes increased shear force during exercise (for expelling lymphocytes bound to vasculature) and reduced lymphocyte adhesiveness caused by exercise-induced adrenaline production, which causes lymphocyte release from the vascular endothelium, spleen, and lungs [49]. In addition, high-intensity exercise leads to lymphocyte apoptosis, which reduces glutathione levels and mitochondrial transmembrane potential expression but increases phosphatidylserine exposure and DNA fragmentation as well as caspase-3, caspase-8, and caspase-9 activation [50]. Therefore, in the current participants, the counts of lymphocytes and lymphocyte subsets (CD4^+^, CD8^+^, CD19^+^, and CD56^+^) were significantly lower at post-2 than at baseline and post-0, which may be a reason that strenuous endurance exercise induces lymphocyte apoptosis (Figure 4A,B and Figure 5A,B). Additionally, vitamin D can promote responses that amplify innate immune function. The classic VDR is expressed by monocytes as macrophages, but there is a reduction in the expression level of VDR [51]. These changes in VDR are accompanied by the inhibition of satellite cell proliferation and stimulation of differentiation, which may contribute to the maintenance of satellite cell self-renewal capacity. Furthermore, VDR signaling increases mitochondrial biogenesis, while fusion signaling inhibits ROS production, leading to a decrease in antioxidant demand along with a possible increase in competent regenerative phenotype. It has also been reported that within 4 to 24 h following muscle damage, proinflammatory macrophages infiltrate the muscle and secrete cytokines that promote inflammation [2]. Notably, vitamin D_3_ induces myeloid dendritic cells to migrate antigens into various lymphoid tissues and present the antigens to T and B cells, thus enhancing the immune effector response [52]. It has been widely reported that the immunoregulation properties of vitamin D_3_ play a critical role in increased infection susceptibility and inflammatory and immunological disorders [53]. Interestingly, vitamin D_3_ significantly lowers the levels of IL-6, IL1-β, IL-8, and TNF-α, resulting in a decrease in inflammation along with clearance of *Salmonella* colitis infection via autophagic clearance in mice models [54]. In macrophages, 1,25-(OH)_2_D_3_ increases interleukin (IL)-10 and decreases inflammatory stimuli such as tumor necrosis factor-α (TNF-α), receptor activator of nuclear factor kappa-B ligand (RANKL), IL-1β, IL-6, and cyclo-oxygenase-2 (COX-2), resulting in anti-inflammatory activity [55]. Vitamin D may exert anti-inflammatory characteristics by upregulating mitogen-activated protein kinase (MAPK) phosphatase (MKP)-1 and inhibiting p38 activation and cytokine production in monocytes/macrophages [56]. Recent studies have described 1,25D-mediated triggering of receptors on myeloid cells-1 (TREM-1) [57], a cell-surface protein associated with cytokine and chemokine production [58] that can amplify toll-like receptor (TLR) signaling. The immunomodulatory effects of vitamin D also include reduced inflammatory cytokines release, such as IL-1β via inflammasome activation [59]. Additionally, the complex of calcitriol, VDR, and retinoid X receptor directly activates the transcription of antimicrobial peptides such as defensin β2 and cathelicidin antimicrobial peptide [60,61,62]. Besides toll-like receptor signaling, other cytokines such as interferon-γ or IL-4 have also been found to affect CYP27B1 expression, which provides instructions to synthesize 1α-hydroxylase enzyme to activate vitamin D_3_ [63].

We also observed that CD19^+^ counts in all participants were significantly lower at post-2 than at baseline (Figure 5A). This result differs from that of another study indicating that endurance exercise does not alter CD19^+^ counts [11]. We speculate that this dissimilarity is due to the difference among average VO_2max_ value (66.5 ± 5.3 mL/kg/min) in the study of Shek et al. [11] and vitamin D_3_ and placebo groups of our study, with values being 47.89 ± 8.81 and 44.78 ± 6.26 mL/kg/min, respectively [11]. Therefore, differences in aerobic capacity may explain how endurance exercise triggers CD19^+^ count suppression. In addition, CD56^+^ is an innate immune cell type with a vigorous cytolytic function against virus-infected cells [64]. Here, we observed that the changes in CD56^+^ count in all the participants were significantly higher at post-0 than at baseline (Figure 5B). By contrast, a study reported that CD56^+^ counts in all participants performing 2 h of cycling exercise were significantly lower 8 h after exercise than before exercise [5]. We speculate that this difference may be at least partly due to a difference in participant selection by Kakanis et al. [5], with recruitment of elite athletes who exercised at an intensity of 90% second ventilator threshold (70–80% VO_2max_). Whereas, in our study, healthy men were recruited who exercised at 65% VO_2max_ intensity. Furthermore, CD8^+^ counts in both the groups were significantly higher at post-0 (Figure 4B), but the CD4^+^/CD8^+^ ratio was significantly lower at post-0 (Figure 4C), possibly because CD8^+^ contains a higher density of β_2_-adrenoceptors than does CD4^+^, which results in increased CD8^+^ counts and thus a decreased CD4^+^/CD8^+^ ratio [65]. In addition, the CD4^+^/CD8^+^ ratio has been demonstrated to be an effective parameter for assessing immune function [66,67]. When the CD4^+^/CD8^+^ ratio is <1.0, T-cell proliferation decreases, and T-cell mortality increases [68]. However, the present study revealed insignificant between-group differences in the CD4^+^/CD8^+^ ratio at any time point. However, in the placebo group, the CD4^+^/CD8^+^ ratio was significantly lower at post-4 and post-24 (1.05 ± 0.33 and 1.04 ± 0.29, respectively) than at baseline (Figure 4C). By contrast, the differences in the CD4^+^/CD8^+^ ratio were insignificant in the vitamin D_3_ groups between any time point after exercise and at baseline, and the CD4^+^/CD8^+^ ratios at post-4 and post-24 were 1.18 ± 0.31 and 1.12 ± 0.21, respectively (Figure 4C). Therefore, in the present study, vitamin D_3_ supplementation demonstrates potential benefits on immune response after strenuous endurance exercise. Studies should evaluate the efficiency of different vitamin D_3_ supplementation in a dose- and time-dependent fashion.

In the present study, TNF-α levels in the vitamin D_3_ group were significantly higher at post-0 than at baseline, and they reverted to the baseline levels at post-2. However, the TNF-α level in the placebo group remained higher after exercise than before exercise for 4 h, indicating that vitamin D_3_ potentially enhances recovery to baseline levels of TNF-α (Figure 6A). In addition, IL-6 levels in the vitamin D_3_ group at post-2, post-4, and post-24 were significantly lower than that in the placebo group (Figure 6B). Similarly, a study indicated that increasing blood 25(OH)D levels effectively attenuates TNF-α and IL-6 levels after strenuous endurance exercise [36]. Further, 1,25(OH)_2_D_3_ has also been noted to inhibit proinflammatory cytokine expression, which is consistent with our results [69,70,71]. In SD rats, high-intensity exercise-induced muscle damage has been shown to activate p38 MAPK, which might promote nuclear translocation of p65 NF-κB in addition to expression of TNF-α and IL-6 gene [72]. This study further demonstrated that vitamin D_3_ supplementation may be advantageous in the accelerated recovery or in rescuing muscle damage. However, no altered inflammatory biomarker profile (TNF-α and IL-6) has been reported on vitamin D_3_ (4000 IU) supplementation during exercise training [73], whereas another study showed no significant effect of vitamin D_3_ (10,000 IU) supplementation per day for 2 weeks on IL-6, and the 25(OH)D levels in the participants remained <30 ng/mL [37]. On the contrary, our participants consumed 5000 IU of vitamin D_3_ daily for 4 weeks, and their 25(OH)D levels increased to 44.67 ± 6.89 ng/mL (Figure 2). Therefore, post-supplementation 25(OH)D levels may be a factor determining whether exercise increases IL-6 levels. Although we did not differentiate between the pro-inflammatory macrophage-derived and the anti-inflammatory muscle-derived IL-6, in addition to our opinion, a previous study suggests that during physical exercise, muscle fibers generate IL-6 via a pathway that is independent of TNF-α [74]. Further, IL-6 prompts the release of other anti-inflammatory cytokines such as IL-1ra and IL-10 into the bloodstream while simultaneously impeding the production of the pro-inflammatory cytokine TNF-α. Moreover, IL-6 boosts lipid metabolism by promoting lipolysis and glycogenolysis [75]. Based on these observations, we propose that secretion of IL-6 may be related to anti-inflammatory-promoting activity and energy metabolism. In addition, the relationship between vitamin D consumption and exercise-induced muscle damage has been inconsistent. In some reports, such as Roca et al.’s study, the biomarkers of muscle damage peaked at 48 h after exercise [76], but in the present study, following many other previous studies, measurements were taken up to 24 h after exercise [77,78,79]. However, future studies are necessary to measure muscle damage biomarkers longer than 24 h.

In a study, participants consumed vitamin D_3_ (1000 IU/day for 12 weeks) in tablet form, and the results demonstrated decreased IL-1β and myoglobin levels without, however, affecting CK activity [38]. Another report in which participants consumed vitamin D_2_ (600 IU/day) in powder form revealed no effects on muscular function or exercise-induced muscle damage [39]. In contrast, the participants of our study consumed vitamin D_3_ (5000 IU/day for 4 weeks) in oil form and demonstrated increased vitamin D_3_ absorption and high blood 25(OH)D levels, which appear to be related to the reduction in muscle damage biomarker levels (CK and LDH). Therefore, considering differences in the bioavailability of vitamin D_3_ in the solid and oil forms, vitamin D_3_ supplementation in the oil form at 5000 IU/day for 4 weeks may be a practical strategy for alleviating exercise-induced muscle damage in endurance athletes. Vitamin D_3_ might elevate the strength and performance of skeletal muscle tissue [80], perhaps via sensitization of the calcium-binding sites on the sarcoplasmic reticulum, resulting in increased muscle contractions [81]. Of note, mixed results have been evidenced on the use of vitamin D supplements, with a positive impact on muscular function observed only in participants having insufficient status (25(OH)D < 50 nmol/l) [82]. Vitamin D is also associated with the skeletal muscle regeneration process, as the secosteroid (fat-soluble secosteroid prohormone) seems to exhibit an ability to mediate myoblast proliferation and differentiation [83]. Vitamin D enhances calcium absorption and bone mineralization, boosts muscle function, and is important for musculoskeletal health [84]. Recently, it was revealed that 25(OH)D insufficiency diminishes signaling pathways such as MAPK and AKT that are responsible for growth and survival and weakens muscle cell development [85]. However, in contrast to our results, a systematic review and meta-analysis documented no significant effects of vitamin D on CK, LDH, and myoglobin during post-exercise muscle recovery [86]. Further, it is known that skeletal muscles receive a large fraction of blood and oxygen due to increased heart rate after aerobic exercise. AHR and HR_max_ are generally used as indicators of exercise loading [87]. During exercise, HR_max_ is lower in athletes than in nonathletes [88], and a lower HR_max_ may be associated with higher resting and maximal stroke volumes [89]. Furthermore, an in vivo animal study revealed significantly increased rates of contraction and relaxation in the cardiomyocytes of vitamin D receptor-knockout mice [90].

Our data indicate that HR_max_ and AHR were significantly lower in the vitamin D_3_ group than in the placebo group, but insignificant differences between groups were observed in VO_2max_ and time to exhaustion (Table 2). These results imply that at identical exercise durations and intensities, cardiac output efficiency during exercise is possibly more enhanced by vitamin D_3_ supplementation than by placebo supplementation. A study indicated that IL-6 release increases substrate availability for bioenergetics processes by increasing glycogenolysis and lipolysis in skeletal muscles [91]. In the present study, IL-6 level, AHR, and HR_max_ were significantly lower in the vitamin D_3_ group than in the placebo group. The vitamin D_3_ group required less heart rate output and glycolysis or lipolysis to achieve the same exercise duration and intensity (65% VO_2max_), indicating that daily vitamin D_3_ supplementation at 5000 IU for 4 weeks may also have positive effects on energy utilization. Moreover, studies have reported that 40–47 ng/mL blood 25(OH)D after supplementation significantly and positively increases aerobic capacity and prevents skeletal muscle injuries [36,40]. However, some studies have demonstrated no effects on physical performance [35,92,93]. Furthermore, blood 25(OH)D levels in the participants in other studies were 48–58 ng/mL after supplementation, which demonstrates that vitamin D_3_ supplementation enhanced performance [94,95]. Taken together, our results imply that achieving over 48 ng/mL blood 25(OH)D after 5000 IU vitamin D_3_ supplementation may be a practical strategy to effectively reduce inflammation and muscle damage along with strengthening the immune response. However, additional clinical studies are required to confirm the advantages of our study.

## 5. Conclusions

**Figure 8:**
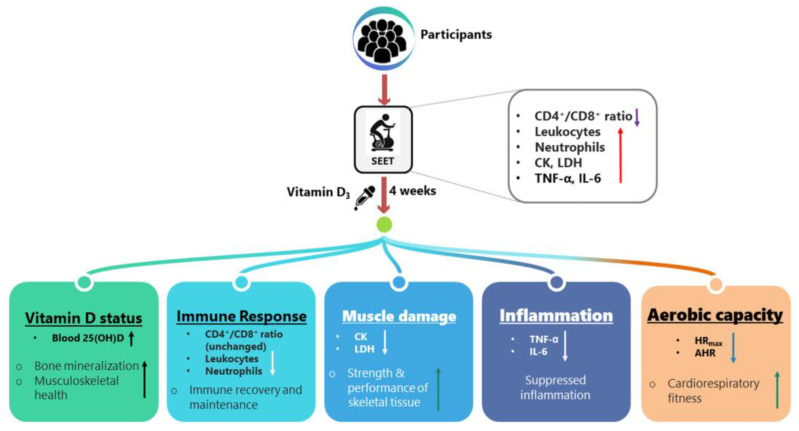
The proposed possible impact of supplemented vitamin D_3_ on immune response, inflammatory cytokines, muscle damage, and aerobic capacity after the strenuous endurance exercise test. CK, creatine kinase; LDH, lactate dehydrogenase; TNF, tumor necrosis factor; IL, interleukin.

Our results imply that daily 5000 IU of vitamin D_3_ supplementation in oil form for 4 weeks effectively exerts positive effects in terms of increased blood 25(OH)D levels and inhibited immune response (CD4^+^/CD8^+^ ratio as well as leukocyte and neutrophil counts), inflammatory cytokines (TNF-α and IL-6), muscle damage (CK and LDH), and increased aerobic capacity (HR_max_ and AHR) (Figure 8).

Taken together, vitamin D_3_ supplementation seems to be an advantageous strategy for the attenuating exercise-induced immune suppression, inflammatory responses, and muscle damage in athletes performing intensive training. Our study also establishes a basis for investigating not only the underlying mechanism of vitamin D_3_-enhanced energy utilization efficiency but also in exploring the effect of vitamin D_3_ supplementation in a dose- and time-dependent fashion as well as their efficiency in terms of cardiac biomarkers and metabolic hormones.

## Figures and Tables

**Table 1:** Participant characteristics.

Variable	Vitamin D_3_ Group	Placebo Group	*p*-Value
Age (years)	21.9 ± 1.4	22.1 ± 2.0	0.784
Body height (cm)	173.1 ± 6.3	173.3 ± 6.8	0.944
Body weight (kg)	64.2 ± 9.1	69.1 ± 8.2	0.248

Data are presented as the mean ± standard deviation (SD); *n* = 9 in each group.

**Table 2:** Changes of aerobic capacity after 4 weeks of vitamin D_3_ supplementation.

Groups	Exhaustion (min)	HR_max_ (beats/min)	AHR (beats/min)	VO_2max_-1 (mL/kg/min)	VO_2max_-2 (mL/kg/min)
Vitamin D_3_	121.48 ± 1.59	176.00 ± 9.06 ^#^	156.89 ± 6.37 ^#^	51.11 ± 5.93	47.89 ± 8.81
Placebo	121.15 ± 0.23	186.22 ± 7.73	167.33 ± 10.31	47.56 ± 5.29	44.78 ± 6.26

Data are presented as mean ± standard deviation (SD); vitamin D_3_ group: *n* = 9. Placebo group: *n* = 9. ^#^ Significant (*p* < 0.05) difference from placebo. Exhaustion, the time to exhaustion; HR_max_, maximal heart rate; AHR, average heart rate; VO_2max_-1, maximal oxygen consumption before supplementation; VO_2max_-2, maximal oxygen consumption after supplementation.

## Data Availability

The data that support the findings of this study are available from the corresponding author upon reasonable request.
